# Anemia in Elderly Patients—The Impact of Hemoglobin Cut-Off Levels on Geriatric Domains

**DOI:** 10.3390/diagnostics13020191

**Published:** 2023-01-05

**Authors:** Francesco Salis, Giambeppe Locci, Barbara Mura, Antonella Mandas

**Affiliations:** 1 Department of Medical Sciences and Public Health, University of Cagliari, 09127 Cagliari, Italy; 2 University Hospital “Azienda Ospedaliero-Universitaria” of Cagliari, 09127 Cagliari, Italy

**Keywords:** Comprehensive Geriatric Assessment (CGA), elderly, anemia, hemoglobin, cut-off levels

## Abstract

Background: The primary aim of this study was to evaluate the impact of anemia—according to the WHO criteria—on cognitive performances, mood, functional and nutritional status, and comorbidities in a population of subjects aged 65 years or older. The secondary aim of this study was to understand if different hemoglobin cut-off levels are associated with a variation of the mentioned domains’ impairment. Methods: We designed a cross-sectional study, including subjects aged 65 or more consecutively evaluated in an outpatient setting from July 2013 to December 2019. A sum of 1698 subjects met the inclusion criteria. They were evaluated with: MMSE and CDT (cognitive assessment), GDS (mood), BADL, IADL, PPT, and POMA (autonomies), MNA (nutritional status), and CIRS (comorbidities). Results: According to the WHO criteria, non-anemic patients reported significantly better performances than the anemics in BADL (*p* < 0.0001), IADL (*p* = 0.0007), PPT (*p* = 0.0278), POMA (*p* = 0.0235), MNA, CIRS TOT, CIRS ICC, and CIRS ISC (*p* < 0.0001). The same tendency has been found by considering the 12 g/dL- and the 13 g/dL-cut-off level in the whole population. The multivariate analysis showed that, considering the 12 g/dL-cut-off level, age (OR: 1.03, *p* = 0.0072), CIRS (OR: 1.08, *p* < 0.0001), and gender (OR: 0.57, *p* = 0.0007) were significant regressors of anemia, while considering the 13 g/dL-cut-off level, age (OR: 1.04, *p* = 0.0001), POMA (OR: 1.03, *p* = 0.0172), MNA (OR = 0.95, *p* = 0.0036), CIRS (OR: 1.17, *p* < 0.0001), ICC (OR = 0.83, *p* = 0.018), and gender (OR = 0.48, *p* < 0.0001) were significant regressors of anemia, while the other CGA variables were excluded by the model (*p* > 0.01). Conclusions: Our study showed that anemia negatively impact on geriatric people’s general status, regardless of which hemoglobin cut-off level is considered. It also highlighted that hemoglobin concentrations < 13 g/dL, regardless of gender, have an association with the impairment of the affective-functional-nutritional state as well as an increase in comorbidities; therefore, it should be pursuable to consider the elderly person “anemic” if Hb < 13 g/dL regardless of gender.

## 1. Background

With the increasing global average age [1,2,3], greater importance is being assigned to geriatric research. It is known that the complexity of this group of the population is well eviscerated by Comprehensive Geriatric Assessment (CGA) [4], which is performed in order to deepen a variety of clinical and socio-environmental domains and their connections [5,6,7]. One of the most common clinical conditions that can be found in elderly people is represented by anemia [8,9,10], which reaches a prevalence of 17% in people at 65 years or more, growing to more than 20% in the oldest [11]. This condition, according to the World Health Organization (WHO), is defined as serum hemoglobin (Hb) levels lower than 12 g/dL in women and 13 g/dL in men [12], regardless of being adult or elderly. In the geriatric population, anemia is often the expression of a multifactorial impairment [10,13,14,15]. Firstly, the aging process is characterized by a low-grade inflammation [16,17], which leads to hepcidin overexpression, with an altered iron regulation, and, consequently, a lower availability for the bone marrow in hemoglobin synthesis [18]. Moreover, elderly people are often malnourished, especially if institutionalized, due to their more compromised status [19]: iron, folates, vitamin B12, and some other nutrients are frequently deficient [8,10,20,21] and such deficiency causes an alteration of erythroid progenitor cells’ cellular cycle [22]. Another crucial contribution is made by chronic kidney disease (CKD) [23]: it is known that the lower the glomerular filtration, the lower the erythropoietin levels [24], but there is another characteristic element contributing to determine anemia, namely the pro-inflammatory status [25], with toxin accumulation, typical of CKD. Lastly, some anemic forms are difficult to classify and are probably determined by a plethora of factors [26,27,28,29], including vascular aging, endocrine disorders, and taking medications. In addition, with the onset of menopause, women have no reason to have increased bleeding than men [30,31,32], likely not justifying a 1 g/dL difference in hemoglobin levels between genders.

Many studies focus on the biochemical and pathophysiological details of anemia’s genesis, while very fewer studies focus on the importance of considering the elderly as a different population than adult [33,34,35,36]: the possible—and frequent—co-existence of so many factors should lead us to ask whether it is correct to continue considering the mentioned hemoglobin cut-off levels even in elderly people or a revision would be helpful [12].

Leading this line, the present study aims to explore the impact of anemia in influencing different domains in a large population aged 65 or more, considering the WHO criteria and also testing other cut-off levels, regardless of gender. 

## 2. Methods

### 2.1. Aim of the Study

The primary aim of this study is to evaluate the impact of anemia—according to the WHO criteria—on cognitive performances, mood, functional and nutritional status, and comorbidities in a population of subjects aged 65 years or older.

The secondary aim of this study is to consider if different hemoglobin cut-off levels are associated with a variation of the mentioned domains’ impairment.

### 2.2. Design of the Study

This cross-sectional study included subjects evaluated at the Geriatric Outpatient Service of the University Hospital of Monserrato, Cagliari, Italy, from July 2013 to December 2019.

### 2.3. Inclusion Criteria

Age ≥ 65 years; having been subjected to CGA; having taken a venous blood sample for blood count on the occasion of the medical examination or less than one week apart.

### 2.4. Exclusion Criteria

Age < 65 years; age ≥ 65 years with acute conditions that contraindicated the CGA’s execution (e.g., psychomotor agitation; delirium; inability to sustain attention); having taken a venous blood sample for blood count more than one week apart from the medical examination. 

A total of 1698 subjects met the inclusion criteria.

## 3. Assessment

The enrolled subjects were evaluated with:

- Mini-Mental State Examination (MMSE) [37] and Clock Drawing Test (CDT) [38], for cognitive assessment;
- Geriatric Depression Scale (GDS) [39], for mood’s assessment;
- Basic Activities of Daily Living (BADL), Instrumental Activities of Daily Living (IADL) [40], Physical Performance Test (PPT) [41], and Performance Oriented Mobility Assessment (POMA) [42], for functional autonomy and physical performance assessment;
- Mini Nutritional Assessment (MNA) [43], for nutritional assessment;
- Cumulative Illness Rating Scale (CIRS) [44], for medical and psychic impairment’s assessment.

The abovementioned tests were administered by trained geriatricians in an outpatient setting.

## 4. Statistical Analysis

The quantitative variables were expressed as median and interquartile ranges (IQR). A Mann–Whitney test was used to compare the continuous variables; a chi-squared test (χ²) was used to compare the qualitative variables. Multivariate analysis was performed with a logistic regression—stepwise (*p*-values > 0.1 were excluded by the model): in order to apply it, the Hb levels were dichotomized such that 1 indicated the presence of anemia. A Kruskal–Wallis’ test was used to perform the analysis of variance; a Conover test was performed for post hoc analysis. The results are reported indicating *p*-values in reference to the 95% confidence intervals (C.I.).

MedCalc software (Version 19.5, Ostend, Belgium) was used for the statistical analysis.

## 5. Results

The study included 1698 subjects aged 65 years or more (median age: 80; ranged from 65 to 100), of whom 1194 (70.3%) were women. The characteristics of the enrolled subjects are shown in Table 1 and Table 2.

From the gender comparison of the population under examination, men achieved significantly better performances than women from a cognitive-affective, functional, and nutritional point of view (MMSE, *p* = 0.0028; CDT, GDS, BADL, PPT, POMA, MNA, *p* < 0.0001). They also showed a higher school education and higher Hb levels (*p* < 0.0001) (Table 3).

The population was divided into two groups based on the presence of anemia—according to the WHO criteria (men, Hb < 13 g/dL; women, Hb < 12 g/dL):

- Anemic group, consisting of 642 patients (37.8% of the sample), of whom 222 males (44% of male population) and 420 females (35.2% of female population);
- Non-anemic group, consisting of 1056 patients (62.2%), of whom 282 males (56%) and 774 females (64.8%).

Table 4 shows the differences between the groups, highlighting that the non-anemic group achieved significantly better performances than the anemic group in BADL (*p* < 0.0001), IADL (*p* = 0.0007), PPT (*p* = 0.0278), POMA (*p* = 0.0235), MNA, CIRS TOT, CIRS ICC, and CIRS ISC (*p* < 0.0001); furthermore, this group was found to be younger (*p* < 0.0001). No difference emerged in the cognitive-affective domain (MMSE, CDT, GDS), nor in BMI.

In order to measure the prevalence of anemia in the two genders, the chi-squared test revealed a higher prevalence of anemia in men (*p* = 0.0006) according to the WHO definition, while the prevalence of anemia was higher in women extending the <12 g/dL and <13 g/dL Hb’s cut-off levels to both males and females (*p* < 0.0001) (Table 5). By applying these Hb’s cut-off levels to the whole study population, we obtained two “new” anemic groups, called: group 1 (Hb < 12 g/dL), consisting of 540 patients (31.8% of the sample), of whom 120 were males (23.8% of male population) and, obviously, 420 females (35.2% of female population), and group 2 (Hb < 13 g/dL), consisting of 979 patients (57.6%), of whom, obviously, 222 were males (44%) and 757 females (63.4%). It goes without saying that we also obtained two “new” non-anemic groups (group 3—Hb ≥ 12 g/dL -, and group 4—Hb ≥ 13 g/dL). From the performed comparison between the new anemic groups and the new non-anemic groups, it emerged that the anemic patients continued to present a more advanced age, a lower level of education, a worse mood, functional and nutritional status, and greater comorbidities both in group 1 vs. group 3 (age, *p* < 0.0001; education, *p* = 0.0012; GDS, *p* = 0.0029; BADL, *p* < 0.0001; IADL, *p* = 0.0104; PPT, *p* = 0.0173; POMA, *p* = 0.0036; MNA, CIRS TOT, CIRS ISC, and CIRS ICC, *p* = 0.0001) (Table 6), and in group 2 vs. group 4 (age, *p* < 0.0001; education, *p* = 0.0007; GDS, *p* = 0.0034; BADL, *p* < 0.0001; IADL, *p* = 0.0075; PPT, *p* = 0.0004; POMA, *p* = 0.0038; MNA, CIRS TOT, CIRS ISC, and CIRS ICC, *p* = 0.0001) (Table 7).

A logistic regression was then used to measure the independent association between the abovementioned variables and Hb levels. A first regression (R1) was applied by using the <12 g/dL cut-off level (Table 8); a second regression (R2) was applied by using the <13 g/dL cut-off level (Table 9). By R1, it emerged that age [Odds Ratio (OR): 1.03, *p* = 0.0072], CIRS TOT (OR: 1.08, *p* < 0.0001), and gender (OR: 0.57, *p* = 0.0007) were significant regressors of anemia, while the other CGA variables were excluded by the model (*p* > 0.01) [Area Under the Curve (AUC): 0.626, 95% Confidence Interval (C.I.): 0.595–0.656, standard error: 0.0183]; by R2, it emerged that age (OR: 1.04, *p* = 0.0001), POMA (OR: 1.03, *p* = 0.0172), MNA (OR = 0.95, *p* = 0.0036), CIRS TOT (OR: 1.17, *p* < 0.0001), CIRS ICC (OR = 0.83, *p* = 0.018), and gender (OR = 0.48, *p* < 0.0001) were significant regressors of anemia, while the other CGA variables were excluded by the model (*p* > 0.01) (AUC: 0.657, 95% C.I.: 0.626–0.686, standard error: 0.0177).

Since age was a significant regressor of Hb, we divided the population in four quartiles according to this variable (Table 10): the median Hb’s levels were 13, 12.7, 12.4, and 12.4 g/dL for first (65–75 years), second (76–80 years), third (81–85 years), and fourth (>85 years) quartiles, respectively. The Conover test (*p* < 0.000001) demonstrated that people in the first quartile had a higher Hb than the second, third, and fourth; the second quartile was higher than the third and fourth, while the third and fourth quartiles did not show significant differences. 

## 6. Discussion

Anemia is the most common hematological disorder in the elderly [8], reaching a prevalence of approximately 17% in people over 65 years of age [11]. The prevalence of anemia is further increased in individuals over 85 years of age [11]. The causes of anemia in the elderly are various and often more than one contributes to its genesis. Anemia has been associated with increased comorbidity (including neurological and psychiatric disorders), worsening physical performance, quality of life, and increased frequency and duration of hospital admissions [45,46,47,48,49,50]. Despite its clinical importance, anemia in the elderly is poorly recognized and the evidence-based guidelines for its management are lacking. Part of the problem is attributable to the definition that is based on WHO criteria established in 1968 since, although most physicians accept this definition, its applicability to elderly patients is questionable [36].

The aim of our study was to evaluate the impact of anemia on cognitive performances, mood, functional and nutritional status, and comorbidities in a population of subjects aged 65 years or older. Secondly, we studied the prevalence of anemia in our population according to the WHO criteria and extending 12 and 13 g/dL Hb’s cut-off levels to the entire population.

Of the 1698 subjects enrolled in this study, 37.8% (642) were anemic according to the WHO criteria, 31.8% (540) considering Hb < 12 g/dL, and 57% (979) considering Hb < 13 g/dL. In the literature, the prevalence of anemia (assessed through the WHO definition) shows a wide variability, ranging from 8% to 25% [11]. The higher prevalence observed in our study could be related to the peculiarity of the service outpatient as it is dedicated to the frail elderly. About etiologies, only a little part of the sample showed a single possible cause of anemia, according to the literature [8].

In our study, the prevalence of anemia was higher in the male gender (44% vs. 35.2%) if the anemia is defined according to the WHO criteria: these findings are consistent with the literature [11]. Nevertheless, a reversal trend was observed—with a higher prevalence in the female gender—both considering Hb < 12 g/dL (35.2% vs. 23.8%) and <13 g/dL (63.4% vs. 44%) cut-off levels. 

Regarding gender, men with anemia presented a better cognitive-affective, functional, and nutritional status than women; these observations are comparable in the gender comparison considering the other Hb’s cut-off levels. The gender comparison—by using all three cut-off levels—in non-anemic subjects showed that men had a better cognitive-affective, functional, and nutritional status than women. As expected [45,46], from our data, it emerged that anemic patients showed a more advanced age, a greater deflection of mood, less functional capacities, malnutrition, and more severe comorbidities. On the contrary, however, we did not observe a greater cognitive impairment in anemic patients, in agreement to some evidence [47]. These aspects were similarly evident according the three cut-off levels, even according to the WHO criteria (but in this case the mood did not show significant differences). 

By studying the independent association between Hb levels and the abovementioned domains, we found that, by considering the <12 g/dL cut-off level, age, comorbidities, and gender were independently associated with anemia, while, by considering the <13 g/dL cut-off level, also worse functional capacities (POMA) and nutritional status were independently associated with anemia.

About age, we found that Hb levels decrease when increasing age, but over the 80 years the difference smooths out, probably owing to the mounting burden affecting very-old people (as showed by CIRS, independently associated with anemia). 

We can therefore assert that hemoglobin concentrations at the lower limits of normal (according to the WHO criteria) have a general negative impact on mood, functional capacity, malnutrition, and comorbidities, but since a worsening of the affective-functional-nutritional status and the comorbidities emerged in subjects with anemia compared to the counterparts without anemia already with Hb < 13 g/dL for both genders, it would be possible to consider this cut-off to identify anemia in the elderly.

## 7. Conclusions

In conclusion, anemia in the elderly, albeit minor, should not be considered as a paraphysiological process of aging, but as an expression of pathology and, as such, worthy of adequate clinical evaluation and treatment. Often, multiple causes contribute to the genesis of anemia in the elderly, justifying the standardization of diagnostic algorithms designed for this category of patients [8]; however, the cut-off levels for anemia defined by the WHO should be redefined for geriatric age. It should be desirable to pay specific attention to hematological statuses in the elderly, since our results showed that anemia negatively impacts different geriatric domains. Moreover, we found that a single cause rarely determines anemia. The raised aspects may lead the physicians to holistically examine the patients, since only a comprehensive and early assessment can sustain the appropriate intervention. First, the causes of anemia, often multiple, have to be recognized and, if possible, fixed. Then, personalized intervention in terms of cognitive, affective, and functional status must be performed.

The present study also highlighted that hemoglobin concentrations < 13 g/dL, regardless of gender, have an association with the impairment of the affective-functional-nutritional state as well as an increase in comorbidities; therefore, it should be productive to consider the elderly person with Hb < 13 g/dL “anemic” regardless of gender, although the recent evidence seems to suggest using other cut-off levels [12,35]. Further studies are necessary to confirm the validity of this hypothesis, relating to hemoglobin levels, clinical outcomes, and pathophysiological aspects in the elderly.

## Figures and Tables

**Table 1:** Characteristics of the sample.

Study Population: n.1698		
Variable	Median	IQR
Age (years)	80	75–85
Education (years)	5	3–8
BMI	26.7	23.5–30.5
MMSE	21.9	16.4–25.7
CDT	4	2–7
GDS	7	4–11
BADL	76	5–92
IADL	2	1–5
PPT	10	7–15
POMA	14	9–20
MNA	21	17.5–23
CIRS TOT.	31	28–34
CIRS ISC	2.2	2–2.4
CIRS ICC	7	5–8
Hb	12.6	11.6–13.7

IQR: interquartile range; BMI: Body Mass Index; MMSE: Mini Mental State Examination; CDT: Clock Drawing Test; GDS: Geriatric Depression Scale; BADL: Basic Activities of Daily Living; IADL: Instrumental Activities of Daily Living; PPT: Physical Performance Test; POMA: Performance Oriented Mobility Assessment; MNA: Mini Nutritional Assessment; CIRS: Cumulative Illness Rating Scale; CIRS ICC: Comorbidity Index Score; CIRS ISC: Severity Index; CIRS TOT.: Total score; Hb: hemoglobin.

**Table 2:** Hematological Conditions.

Hematological Condition	Percentage
History of leukemia	1.5%
Severe Chronic Kidney Disease	4%
Iron Deficiency ^a^	9.5%
Folate Deficiency ^b^	17.1%
Vitamin B12 Deficiency ^c^	6.2%

^a^, data available in 1035 subjects; ^b^, data available in 1022 subjects; ^c^, data available in 1028 subjects.

**Table 3:** Gender comparison.

	Men n.504			Women n.1194			Mann–Whitney Test
	Median	Min–Max	IQR	Median	Min–Max	IQR	*p*-Value
Age	80	65–96	74–84.5	80	65–100	75–85	0.0907
Education	5	0–23	5–8	5	0–21	3–5	**<0.0001**
MMSE	23	0–30	17–26	21.5	0–30	16–25	**0.0028**
CDT	5	0–9	3–8	3	0–9	2–6	**<0.0001**
GDS	5	0–15	3–8	8	0–15	5–11	**<0.0001**
BADL	83	1–100	63–96.5	74	0–100	55–90	**<0.0001**
IADL	2	0–8	1–5	2	0–8	1–5	0.3486
PPT	11	0–27	7–17	9	0–28	6–14	**<0.0001**
POMA	16	0–28	11–22	13	0–28	9–19	**<0.0001**
MNA	21.25	2–29	18–24.5	20.5	2–30	17.5–23	**<0.0001**
BMI	26.45	15–51	23.5–29	26.9	15–48	23.5–31	**0.0412**
CIRS TOT.	32	15–45	28–35	31	5–45	28–34	0.0573
CIRS ISC	2.23	1–3	2–2.5	2,21	1–3	2–2.4	0.0577
CIRS ICC	7	0–12	5–8	7	1–13	5–8	0.3524

Min: minimum; Max: maximum; IQR: interquartile range; BMI: Body Mass Index; MMSE: Mini Mental State Examination; CDT: Clock Drawing Test; GDS: Geriatric Depression Scale; BADL: Basic Activities of Daily Living; IADL: Instrumental Activities of Daily Living; POMA: PPT: Physical Performance Test; Performance Oriented Mobility Assessment; MNA: Mini Nutritional Assessment; CIRS: Cumulative Illness Rating Scale; CIRS ICC: Comorbidity Index Score; CIRS ISC: Severity Index; CIRS TOT.: Total score; Hb: hemoglobin.

**Table 4:** Anemic group vs. non-anemic group (WHO criteria).

	Anemic Group n. 642			Non-Anemic Group n. 1056			Mann–Whitney Test
	Median	Min-Max	IQR	Median	Min-Max	IQR	*p*-Value
Age	81	65–96	77–86	79	65–100	74–84	**<0.0001**
Education	5	0–19	3–8	5	0–23	3–8	0.0587
MMSE	21.7	0–30	16–26	22	0–30	17–26	0.2722
CDT	3	0–9	3–8	4	0–9	2–7	0.2627
GDS	7	0–15	3–8	7	0–15	4–11	0.2487
BADL	73	0–100	63–96.5	78	1–100	59–94	**<0.0001**
IADL	2	0–8	1–5	3	0–8	1–5	**0.0007**
PPT	9	0–27	7–17	10	0–28	7–15	**0.0278**
POMA	14	0–28	11–22	14	0–28	10–21	**0.0235**
MNA	20	6–28.5	18–24.5	21.25	2–30	18–23.5	**<0.0001**
BMI	26.67	15–46	23.5–29	26.9	15–51	24–31	0.2612
CIRS TOT.	32	15–45	28–35	31	16–43	27–34	**<0.0001**
CIRS ISC	2.29	1–3	2–2.5	2.15	1–3	2–2.3	**<0.0001**
CIRS ICC	7	0–13	5–8	6	1–12	5–8	**<0.0001**

Min: minimum; Max: maximum; IQR: interquartile range; BMI: Body Mass Index; MMSE: Mini Mental State Examination; CDT: Clock Drawing Test; GDS: Geriatric Depression Scale; BADL: Basic Activities of Daily Living; IADL: Instrumental Activities of Daily Living; PPT: Physical Performance Test; POMA: Performance Oriented Mobility Assessment; MNA: Mini Nutritional Assessment; CIRS: Cumulative Illness Rating Scale; CIRS ICC: Comorbidity Index Score; CIRS ISC: Severity Index; CIRS TOT.: Total score; Hb: hemoglobin.

**Table 5:** Prevalence of anemia according to different cut-off levels.

	Men	Women	χ^2^ (*p*-Value)
WHO Criteria	222 (44%)	420 (35.2%)	**0.0006**
Cut-off Hb < 12 g/dL	120 (23.8%)	420 (35.2%)	**<0.0001**
Cut-off Hb < 13 g/dL	222 (44%)	757 (63.4%)	**<0.0001**

WHO: World Health Organization; Hb: hemoglobin; χ^2^: chi-squared test.

**Table 6:** Anemic group vs. non-anemic group (cut-off: Hb < 12 g/dL).

	Group 1 (Hb < 12 g/dL) n. 540		Group 3 (Hb ≥ 12 g/dL) n. 1158		Mann–Whitney Test
	Median	IQR	Median	IQR	*p*-Value
Age	82	77–86	79	74–84	**<0.0001**
Education	5	3–6,5	5	3–8	**0.0012**
MMSE	21.4	15.65–25.7	22	16.7–25.7	0.0692
CDT	3	2–7	4	2–7	0.0863
GDS	8	5–11	7	3.5–11	**0.0029**
BADL	73	54–88	78	59–94	**<0.0001**
IADL	2	1–4	2	1–5	**0.0104**
PPT	9	6–14	10	7–15	**0.0173**
POMA	13	9–19	14	10–21	**0.0036**
MNA	19.75	17–22.5	21	18–23.5	**<0.0001**
BMI	26.6	23.44–30.51	26.9	23.63–30.61	0.3676
CIRS TOT.	32	29–35	31	28–34	**<0.0001**
CIRS ISC	2.29	2.07–2.5	2.19	1.93–2.38	**<0.0001**
CIRS ICC	7	5–8	6	5–8	**<0.0001**

IQR: interquartile range; BMI: Body Mass Index; MMSE: Mini Mental State Examination; CDT: Clock Drawing Test; GDS: Geriatric Depression Scale; BADL: Basic Activities of Daily Living; IADL: Instrumental Activities of Daily Living; PPT: Physical Performance Test; POMA: Performance Oriented Mobility Assessment; MNA: Mini Nutritional Assessment; CIRS: Cumulative Illness Rating Scale; CIRS ICC: Comorbidity Index Score; CIRS ISC: Severity Index; CIRS TOT.: Total score; Hb: hemoglobin.

**Table 7:** Anemic group vs. non-anemic group (cut-off: Hb < 13 g/dL).

	Group 2 (Hb < 13 g/dL) n. 979		Group 4 (Hb ≥ 13 g/dL) n. 719		Mann–Whitney Test
	Median	IQR	Median	IQR	*p*-Value
Age	81	76–86	79	74–83	**<0.0001**
Education	5	3–7	5	4–8	**0.0007**
MMSE	21.7	16.2–25.7	22.35	16.7–25.7	0.2832
CDT	3	2–7	4	2–7	0.1429
GDS	8	4–11	7	3–11	**0.0034**
BADL	73	55–90	81	61–95	**<0.0001**
IADL	2	1–5	3	1–5	**0.0075**
PPT	9	6–14	11	7–16	**0.0004**
POMA	14	9–19	15	10–22	**0.0038**
MNA	20	17–22.5	21.5	18.5–24	**<0.0001**
BMI	26.67	23.44–30.49	27.03	23.8–30.71	0.2231
CIRS TOT.	32	29–35	30	27–33	**<0.0001**
CIRS ISC	2.29	2–2.46	2.14	1.93–2.36	**<0.0001**
CIRS ICC	7	5–8	6	5–8	**<0.0001**

IQR: interquartile range; BMI: Body Mass Index; MMSE: Mini Mental State Examination; CDT: Clock Drawing Test; GDS: Geriatric Depression Scale; BADL: Basic Activities of Daily Living; IADL: Instrumental Activities of Daily Living; PPT: Physical Performance Test; POMA: Performance Oriented Mobility Assessment; MNA: Mini Nutritional Assessment; CIRS: Cumulative Illness Rating Scale; CIRS ICC: Comorbidity Index Score; CIRS ISC: Severity Index; CIRS TOT.: Total score; Hb: hemoglobin.

**Table 8:** Logistic regression stepwise Hb < 12 g/dL.

Variable *	Coefficient	Std. Error	OR	95% C.I.	*p*-Value
Age	0.028	0.01	1.03	1.01–1.05	**0.0072**
CIRS TOT	0.078	0.016	1.08	1.05–1.11	**<0.0001**
Gender	−0.553	0.162	0.57	0.42–0.79	**0.0007**

* MMSE, CDT, GDS, BADL, IADL, PPT, POMA, MNA, CIRS ICC, CIRS ISC, BMI, and education were excluded by the model (*p* > 0.01) OR: odds ratio; C.I.: Confidence Interval; BMI: Body Mass Index; MMSE: Mini Mental State Examination; CDT: Clock Drawing Test; GDS: Geriatric Depression Scale; BADL: Basic Activities of Daily Living; IADL: Instrumental Activities of Daily Living; PPT: Physical Performance Test; POMA: Performance Oriented Mobility Assessment; MNA: Mini Nutritional Assessment; CIRS: Cumulative Illness Rating Scale; CIRS ICC: Comorbidity Index Score; CIRS ISC: Severity Index; CIRS TOT.: Total score; Hb: hemoglobin.

**Table 9:** Logistic regression stepwise Hb < 13 g/dL.

Variable *	Coefficient	Std. Error	OR	95% C.I.	*p*-Value
Age	0.042	0.01	1.04	1.02–1.06	**0.0001**
POMA	0.029	0.012	1.03	1.005–1.054	**0.0172**
MNA	−0.055	0.018	0.95	0 91–0.98	**0.0036**
CIRS TOT	0.154	0.037	1.17	1.08–1.25	**<0.0001**
CIRS ICC	−0.185	0.074	0.83	0.72–0.96	**0.0128**
Gender	−0.726	0.152	0.48	0.36–0.65	**<0.0001**

* MMSE, CDT, GDS, BADL, IADL, PPT, CIRS ISC, BMI, and education were excluded by the model (*p* > 0.01) OR: odds ratio; C.I.: Confidence Interval; BMI: Body Mass Index; MMSE: Mini Mental State Examination; CDT: Clock Drawing Test; GDS: Geriatric Depression Scale; BADL: Basic Activities of Daily Living; IADL: Instrumental Activities of Daily Living; PPT: Physical Performance Test; POMA: Performance Oriented Mobility Assessment; MNA: Mini Nutritional Assessment; CIRS: Cumulative Illness Rating Scale; CIRS ICC: Comorbidity Index Score; CIRS ISC: Severity Index; CIRS TOT.: Total score; Hb: hemoglobin.

**Table 10:** Kruskal–Wallis and Conover Test.

Quartile	Median	Average Rank	Different From
1 (65–75 years)	13	958.83	2, 3, and 4
2 (76–80 years)	12.7	869.56	1, 3, and 4
3 (81–85 years)	12.4	796.63	1 and 2
4 (>85 years)	12.4	750.59	1 and 2

## Data Availability

The data and materials used and/or analyzed during the current study are not publicly available. The are available from the corresponding author upon reasonable request.

## Abbreviations

BMI: Body Mass Index; Comprehensive Geriatric Assessment (CGA), MMSE: Mini Mental State Examination; CDT: Clock Drawing Test; GDS: Geriatric Depression Scale; BADL: Basic Activities of Daily Living; IADL: Instrumental Activities of Daily Living; POMA: PPT: Physical Performance Test; Performance Oriented Mobility Assessment; MNA: Mini Nutritional Assessment; CIRS: Cumulative Illness Rating Scale; CIRS ICC: Comorbidity Index Score; CIRS ISC: Severity Index; CIRS TOT.: Total score; Hb: hemoglobin.
